# Comprehensive Analysis of Molecular Clusters and Prognostic Signature Based on m7G-related LncRNAs in Esophageal Squamous Cell Carcinoma

**DOI:** 10.3389/fonc.2022.893186

**Published:** 2022-07-14

**Authors:** Fangchao Zhao, Zefang Dong, Yishuai Li, Shiquan Liu, Pengfei Guo, Dengfeng Zhang, Shujun Li

**Affiliations:** ^1^ Department of Thoracic Surgery, The Second Hospital of Hebei Medical University, Shijiazhuang, China; ^2^ School of Clinical Medicine, Hebei Medical University, Shijiazhuang, China; ^3^ Department of Thoracic Surgery, Hebei Chest Hospital, Shijiazhuang, China; ^4^ Department of Thoracic Surgery, Affiliated Hospital of Chengde Medical University, Chengde, China

**Keywords:** esophageal squamous cell carcinoma, N7-methylandenosine, long non-coding RNA, prognostic signature, immune microenvironment

## Abstract

N7-Methylguanosine (m7G) and long non-coding RNAs (lncRNAs) have been widely reported to play an important role in cancer. However, there is little known about the relationship between m7G-related lncRNAs and esophageal squamous cell carcinoma (ESCC). In this study, we aimed to find new potential biomarkers and construct an m7G-related lncRNA prognostic signature for ESCC. Three molecular clusters were identified by consensus clustering of 963 m7G-related lncRNAs, of which cluster B is preferentially related to poorer prognosis, higher immune and stromal scores, higher mRNA levels of immune checkpoints, and higher immune infiltrate level. We constructed a robust and effective m7G-related lncRNA prognostic signature (m7G-LPS, including 7 m7G-related prognostic lncRNAs) and demonstrated its prognostic value and predictive ability in the GEO and TCGA cohorts. The risk score was able to serve as an independent risk factor for patients with ESCC and showed better prediction than the traditional clinical risk factors. The immune score, stromal score, the infiltration level of immune cells and expression of immune checkpoints were significantly higher in the high-risk subgroup compared to the low-risk subgroup. The establishment of nomogram further improved the performance of m7G-LPS and facilitated its clinical application. Finally, we used GTEx RNA-seq data and qRT-PCR experiments to verify the expression levels of 7 m7G-related lncRNAs. To a certain degree, m7G-lncRNAs can be used as prognostic markers and therapeutic targets for ESCC patients.

## Introduction

In 2020, there were 604,000 new cases of esophageal cancer and 544,000 deaths worldwide, ranking 7th and 6th in incidence and mortality of malignant tumors, respectively (1). The number of esophageal cancer cases and deaths in China and their global percentages were 324,000 (53.7%) and 301,000 (55.3%), respectively (2). Esophageal cancer is more malignant, prone to recurrence and metastasis, and has a poor prognosis (3). There are two main histological types of esophageal cancer: esophageal squamous cell carcinoma (ESCC) and esophageal adenocarcinoma, of which ESCC is the common histological type of esophageal cancer in China, accounting for more than 80% (4). Despite the increasing maturity of esophageal cancer treatment technology, the overall 5-year survival rate is still below 20% (5). Therefore, reliable predictive biomarkers and the according predictive model are essential for early precise diagnosis and individualized treatment for ESCC patients.

N7-Methylguanosine (m7G) is a modification in which a methyl group is added to the seventh N position of the messenger RNA guanine (G) by the action of methylation transferase. m7G modification is one of the most common forms of base modification in post-transcriptional regulation and is widely distributed in the 5’ hat region of tRNA, rRNA, mRNA and lncRNA, which is important for maintaining RNA processing metabolism, stabilization, nuclear export and protein translation (6, 7). Recent studies have shown that m7G modification regulates oncogenesis and progression. For example, METTL1-mediated m7G tRNA modification regulates the translation of EGFR/EFEMP1 and finally promotes the bladder cancer development (8). Additionally, N7-methylguanosine tRNA modification promotes tumorigenesis and chemoresistance through WNT/β-catenin pathway in nasopharyngeal carcinoma (9). However, the specific role of m7G modification in lncRNAs remains unclear; therefore, understanding the mechanism of m7G-related lncRNAs in the development of ESCC may be useful for prognostic targets.

In the present study, we systematically explored the prognostic significance and tumor microenvironment (TME) heterogenicity of m7G-lncRNAs in ESCC. This study may provide new guidance on survival outcomes and treatment strategies for ESCC.

## Materials and Methods

### Datasets and Pre-Processing

The RNA-seq transcriptome data [fragments per kilobase million (FPKM)] and corresponding clinical data of ESCC patients were extracted from TCGA database (ESCC tissue samples: 80, normal epithelial tissue samples: 11) and GEO database (GSE53624, ESCC tissue samples: 119). In each data set, duplicate sequencing samples from the same patient were excluded, and patients lacking complete follow-up information and with zero survival days were excluded. In addition, copy number variation (CNV) and somatic mutation data of ESCC were downloaded from the TCGA database. It is worth mentioning that after TPM conversion of RNA-Seq data in FPKM format, background correction, normalization and expression calculation were performed using the “sva” package to remove batch effects. The m7G-related genes were obtained from the literature (6) and the gene sets GOMF_M7G_5_PPPN_DIPHOSPHATASE_ACTIVITY,GOMF_RNA_CAP_BINDING, and GOMF_RNA_7_METHYLGUANOSINE_CAP_BINDING. In TCGA cohort, we can annotated 24 m7G-related genes, including DCP2, IFIT5, EIF3D, EIF4G3, NSUN2, GEMIN5, AGO2, EIF4E, EIF4E2, NCBP2, NUDT11, NUDT3, NCBP1, METTL1, LARP1, NUDT4, SNUPN, WDR4, LSM1, NUDT16, CYFIP1, NUDT10, EIF4E3, and DCPS. However, three m7G-related genes were excluded from meta dataset: NUDT10, EIF4E3, and DCPS. Spearman correlation analysis was performed on all lncRNAs and 21 m7G-related genes (*p* < 0.001, correlation coefficient > 0.35). Finally, 963 m7G-related lncRNAs were screened for subsequent bioinformatics analysis.

### Evaluation of the Immune Microenvironment

The ssGSEA score (xi) was calculated for each ESCC sample (i) using the ssGSEA algorithm, transformed using the formula xi = (xi-xmin)/(xmax-xmin). where xmax and xmin represent the maximum and minimum ssGSEA scores of all samples in the ESCC dataset, respectively. This same method was applied to calculate the scores of 29 immune cells and immune functions in ESCC samples, and the “pheatmap” package was used to visualize the heat map. In addition, the ESTIMATE algorithm was applied to identify specific features associated with stromal cell and immune cell infiltration. Differences between different clusters were compared using Kruskal Wallis test.

### Unsupervised Clustering

Unsupervised consistent clustering analysis was performed based on the expression levels of m7G-related lncRNAs. The number of clusters was determined using the R package “conensusClusterPlus” and 100 replicates with pltem = 0.8 were performed to verify the stability of the clusters. Kaplan-Meier curve was used to assess the overall survival time (OS) of different ESCC patients in the dataset. Principal component analysis (PCA) analysis was used to determine the ability of molecular clusters or risk groupings to discriminate between patients with a dimensionality reduction method.

### Construction and Validation of m7g-related LncRNA Prognostic Signature (m7G-LPS)

In the batch-corrected GEO cohort, m7G-related prognostic lncRNAs were identified by univariate Cox regression analysis (*p* < 0.01). Subsequently, the least absolute shrinkage and selection operator (LASSO) regression analysis was applied to eliminate those redundant lncRNAs. Based on the coefficients of multivariate Cox regression, a m7G-LPS was constructed and the TCGA cohort was used as an external validation set for the validation of predictive efficacy. The receiver operating characteristic curves (ROC) curve analysis was performed by “timeROC” package. Independent prognostic factors determined by multivariate Cox regression were used to construct nomogram, and the accuracy of the nomogram was verified using calibration curves.

### Enrichment Analysis

The gene set variation analysis (GSVA) was used to assess differences in biological pathways between clusters. Gene Ontology (GO) is used to annotate the biological processes, molecular functions, and cellular components of genes. Gene pathways were annotated using the Kyoto Encyclopedia of Genes and Genomes (KEGG). Differential genes between different clusters were analyzed using the “limma” package (*p* < 0.001). Overlapping genes between the three groups were then subjected to GO and KEGG analysis using the “clusterProfiler” package. In addition, c2.cp.kegg.v7.0.symbols.gmt was used as the reference gene set, with FDR < 0.05 as the screening threshold.

### Drug Sensitivity Analysis

The half-maximal inhibitory concentration (IC50) was calculated using the “prophetic” package in R software. Chemotherapy drugs were obtained from the genome of Drug Sensitivity in Cancer (GDSC) database.

### Tissue Samples and Quantitative Real-time Polymerase Chain Reaction

A total of 8 matched tumor/normal tissue samples were collected from patients with ESCC in the operating room of the Second Hospital of Hebei Medical University (Shijiazhuang, China). The inclusion criteria of tissue specimens included as follows: (1) pathological diagnosis for ESCC; (2) except ESCC with no other malignancy; (3) consistent with radical esophageal cancer surgery indications; (4) no radiotherapy and chemotherapy before surgery. The study was approved by the Ethics Committee of the Second Hospital of Hebei Medical University (No. 2022-R241) and informed written consent was obtained from all patients. The specific experimental protocol comes from our previous research (10, 11).

### Statistical Analysis

All the statistical analyses were made with the R software (v.4.0.1). The above section has described detailed statistical approaches for transcriptome data processing. A p-value less than 0.05 was of statistical significance.

## Results

### Landscape of m7G Regulators in ESCC

**Figure 1:**
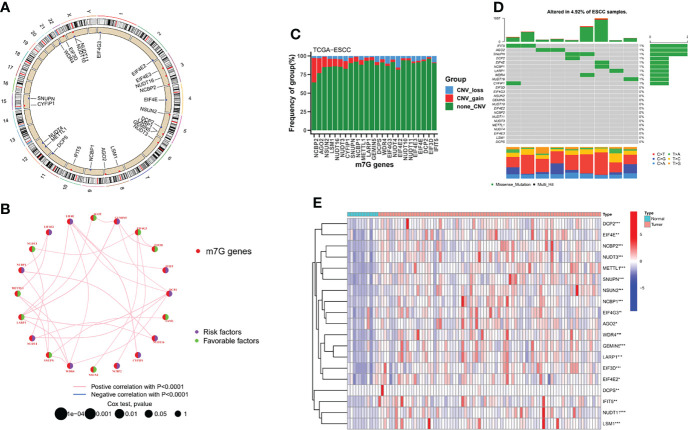
Landscape of m7G regulators in ESCC. **(A)** Location of the 21 m7G regulators in the chromosome. **(B)** Interactions among m7G regulators in ESCC. **(C)** The CNV variation frequency of m7G regulators in the TCGA-ESCC cohort. **(D)** The mutation frequency and classification of m7G regulators. **(E)** Heatmap indicated the differences in the expression of m7G regulators in normal and tumor tissues. *P < 0.05; **P < 0.01; ***P < 0.001.

The overall workflow of the current study is displayed in **Figure S1**. The location on chromosomes and expression level of the 21 annotatable m7G regulators were illustrated by a circos plot (**Figure 1A**). The regulatory network depicted the correlation network of m7G regulators, regulator relationships, and their survival significance in ESCC patients (**Figure 1B**).We found a strong association between most of the m7G regulators. Nine of these regulators could be used as protective factors, 11 regulators were risk factors, and only one regulator had no prognostic significance and was not shown in the figure. In addition, we found that the highest amplification frequency was present in NCBP2, while the highest deletion frequency was present in EIF4E2 (**Figure 1C**). None of the 21 m7G regulators were found to have significant mutations in the somatic mutation analysis (**Figure 1D**). In addition, we performed a differential analysis of m7G regulators expression in different samples, and the results showed that most of the genes were upregulated in the tumor samples (**Figure 1E**).

### Identification of m7G-related LncRNAs and their Molecular Clusters

**Figure 2:**
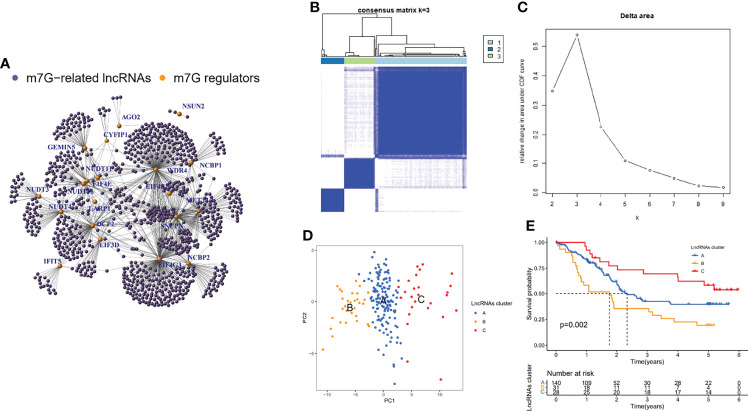
Identification of m7G-related lncRNAs and their molecular clusters. **(A)** Co-expression relationship between m7G-related lncRNAs and m7G regulators. **(B)** Consensus clustering matrix for k = 3. **(C)** Relative change in area under the CDF curve for k = 2 through 9. **(D)** PCA showed that the clusters determined based on the expression of m7G-associated lncRNAs well distinguish ESCC into three clusters. **(E)** Kaplan-Meier curves of OS for three clusters in ESCC.

The GSE35624 cohort and the TCGA cohort were integrated and a total of 963 m7G-related lncRNAs were identified by correlation analysis using the 21 m7G regulators mentioned above and all annotatable lncRNAs (**Figure 2A**). All tumor samples were classified into A/B/C clusters using the R package “ConsensusClusterPlus”. According to the consensus score of the empirical cumulative distribution function (CDF) curve, k = 3 is optimal (**Figures 2B, C**). PCA showed significant heterogeneity in molecular typing based on 963 m7G-related lncRNAs (**Figure 2D**). In addition, survival analysis showed that cluster B had the worst prognosis of these three molecular clusters, while cluster C had the best prognosis (**Figure 2E**).

### Immunological Characterization of m7G-related LncRNA Molecular Clusters

**Figure 3:**
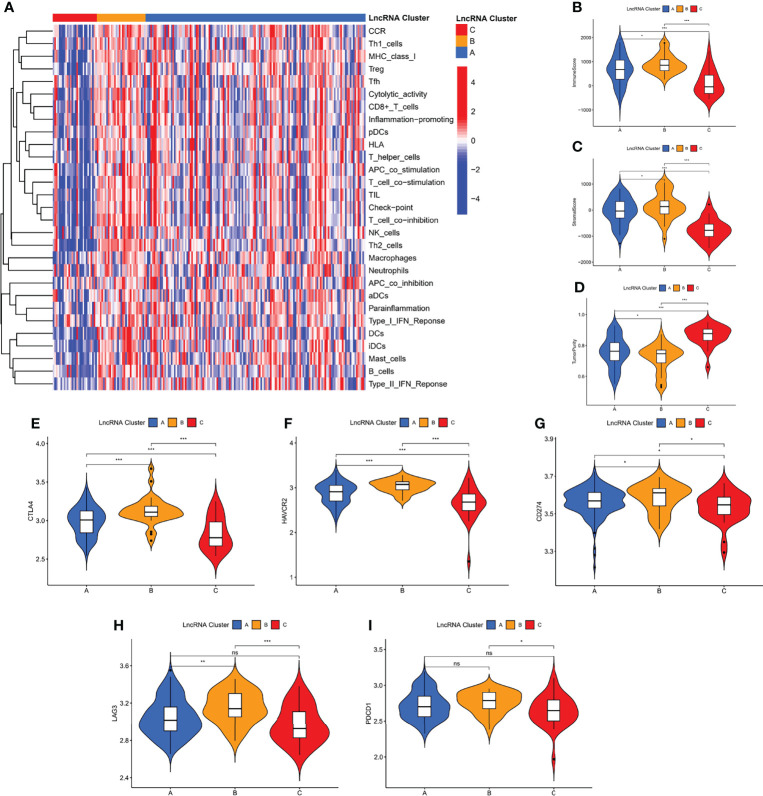
Immunological characterization of m7G-associated lncRNA molecular clusters. **(A)** Heat map of differences in immune cell infiltration between different clusters. The expression level of the immune score **(B)**, stromal score **(C)**, and tumor purity **(D)**, between different clusters. **(E–I)** The expression levels of immune checkpoint genes between different subtypes. *P < 0.05, **P < 0.01, ***P < 0.001, ns, not significant.

Among the three molecular clusters, cluster B had the highest degree of infiltration, followed by clusters A and C (**Figure 3A**). The ESTIMATE algorithm results showed that cluster B had the highest stromal and immune scores and the lowest tumor purity, while cluster A had the lowest stromal and immune scores and the highest tumor purity (stromal and immune scores: cluster B > C > A; tumor purity: cluster A > C > B) (**Figures 3B–D**). Finally, we examined the expression of four immune checkpoint genes (i.e., PDCD1, CTLA4, HAVCR2, LAG3, and CD274) and found that the mRNA levels of immune checkpoints were higher in cluster B than in other clusters, which may suggest that this cluster may benefit more from immunotherapy (**Figures 3E–I**).

### Biological functions of m7G-related LncRNA Molecular Clusters

**Figure 4:**
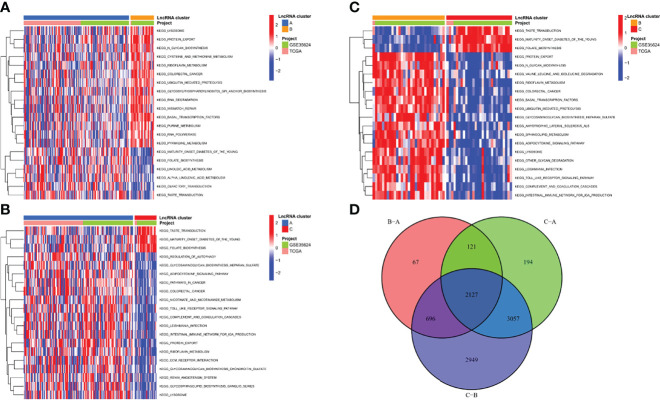
Biological functions of m7G-related lncRNA molecular clusters. **(A–C)** The GSVA pathway enrichment analysis between different subgroups. **(D)** Venn diagram of molecular cluster-associated DEGs.

To investigate the causes of the different survival states and immune landscapes, we used GSVA to study the biological processes between the different clusters. The results showed that cluster B had more activated pathways such as KEGG_LYSOSOME, KEGG_PROTEIN_EXPORT, KEGG_N_GLYCAN_BIOSYNTHESIS, etc. compared with cluster A (**Figure 4A**). Compared with cluster C, cluster A has more activated pathways, such as KEGG_REGULATION_OF_AUTOPHAGY,KEGG_GLYCOSAMINOGLYCAN_BIOSYNTHESIS_HEPARAN_SULFATE,KEGG_ADIPOCYTOKINE_SIGNALING_PATHWAY, etc. (**Figure 4B**). Compared with cluster C, cluster B has more activated pathways, such as KEGG_PROTEIN_EXPORT, KEGG_N_GLYCAN_BIOSYNTHESIS,KEGG_VALINE_LEUCINE_AND_ISOLEUCINE_DEGRADATION, etc. (**Figure 4C**). To further understand the regulatory mechanisms of m7G-associated lncRNA molecular cluster, we identified molecular cluster-associated DEGs, and a total of 2127 genes were considered to be molecular cluster-associated genes (**Figure 4D**).

**Figure 5:**
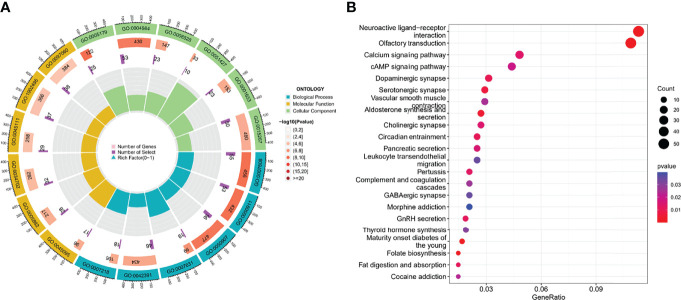
The functional enrichment analysis of cluster-associated DEGs. **(A, B)** GO and KEGG analysis.

GO enrichment analysis showed that they are mainly involved in processes such as sensory perception of smell (GO: 0007608) (**Figure 5A**; **Supplementary File 1**). In the KEGG analysis, the screened DEGs were significantly associated with pathways such as calcium signaling pathway, cAMP signaling pathway (**Figure 5B**; **Supplementary File 2**).

### Construction of the m7G-LPS in the GEO Cohort

**Figure 6:**
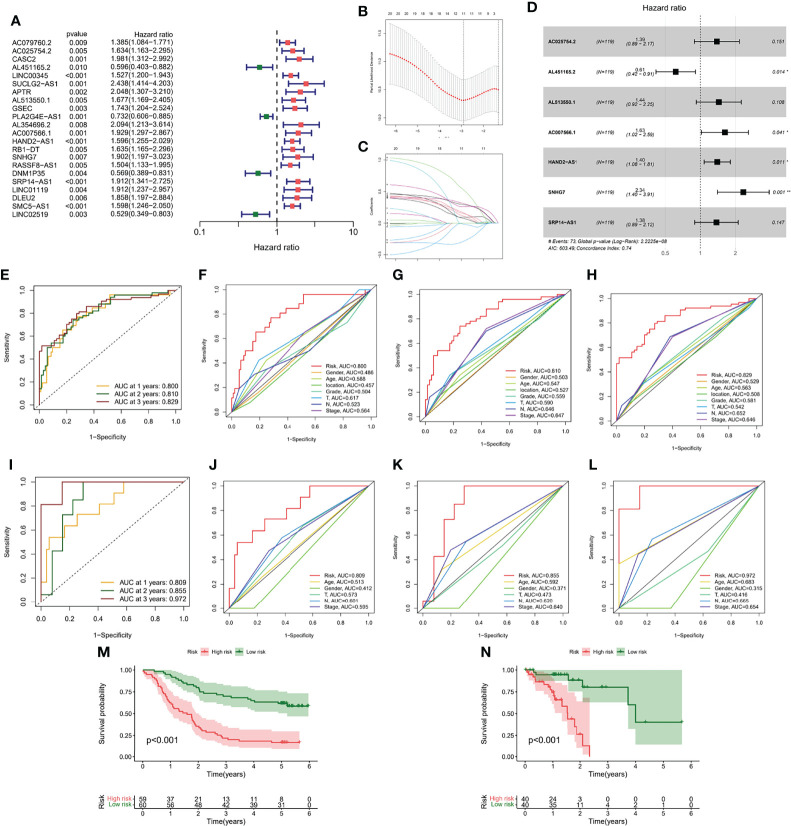
Generation and verification of the m7G-LPS. **(A)** Univariate Cox regression analysis for 22 lncRNAs. **(B)** Cross-validation for tuning the coefficient selection in the LASSO regression. **(C)** LASSO regression of the 22 OS-associated lncRNA. **(D)** Multivariate Cox regression analysis for 7 lncRNAs. ROC curves and their AUC value represented 1-, 2-, and 3-year predictions in the GEO cohort **(E)** and TCGA cohort **(I)**. The 1-year **(F)**, 2-year **(G)** and 3-year **(H)** ROC analysis revealed the AUCs of m7G-related lncRNA risk score and other clinical characteristics in the GEO cohort. The 1-year **(J)**, 2-year **(K)** and 3-year **(L)** ROC analysis revealed the AUCs of m7G-related lncRNA risk score and other clinical characteristics in the TCGA cohort. Kaplan-Meier curve of overall survival of the GEO **(M)** and TCGA cohorts **(N)**.

Although molecular subtyping classification can predict survival and functional differences in ESCC patients, molecular subtyping was studied based on patient populations and therefore cannot accurately predict the survival status of each patient. To facilitate clinical application, we evaluated a single patient based on the expression of m7G-related lncRNAs. Considering the large sample size of the GEO cohort, we screened 22 prognosis-related lncRNAs from the GEO cohort by univariate Cox regression analysis (*p* < 0.05) (**Figure 6A**). Subsequently, we further compressed 22 lncRNAs by LASSO regression analysis (**Figures 6B, C**), and finally obtained 7 lncRNAs with the most prognostic value to build m7G-LPS (**Figure 6D**). The risk score was calculated as follows: risk score = (0.3266 × expression level of AC025754.2) + (-0.4869 × expression level of AL451165.2) + (0.36498 × expression level of AL513550.1) + (0.4860 × expression level of AC007566.1) + (0.3350 × expression level of HAND2-AS1) + (0.8502 × expression level of SNHG7) + (0.3185 × expression level of SRP14-AS1).

### Validation of the m7G-LPS and Construction of the Nomogram

The area under the curve (AUC) of the GEO cohort at 1, 2, and 3 years were 0.800, 0.810, and 0.829, respectively (**Figure 6E**); the AUC at 1, 2, and 3 years were 0.809, 0.855, and 0.972, respectively, in the training cohort (**Figure 6I**). To evaluate the performance of m7G-LPS in predicting the survival of ESCC patients, we performed a comparative analysis of the 1-, 2-, and 3-year AUC values for different clinical indicators. The results found that risk scores in the GEO cohort (**Figures 6F–H**) and TCGA cohort (**Figures 6J–L**) demonstrated better prediction than conventional clinical risk factors (age, gender, T stage, N stage, and TNM stage). Kaplan-Meier curves showed that in the GEO cohort, patients in the high-risk subgroup had significantly lower survival time and survival rate than the low-risk subgroup (*p* < 0.001) (**Figure 6M**), which was also verified in samples from the TCGA cohort (*p* < 0.001) (**Figure 6N**).

**Figure 7:**
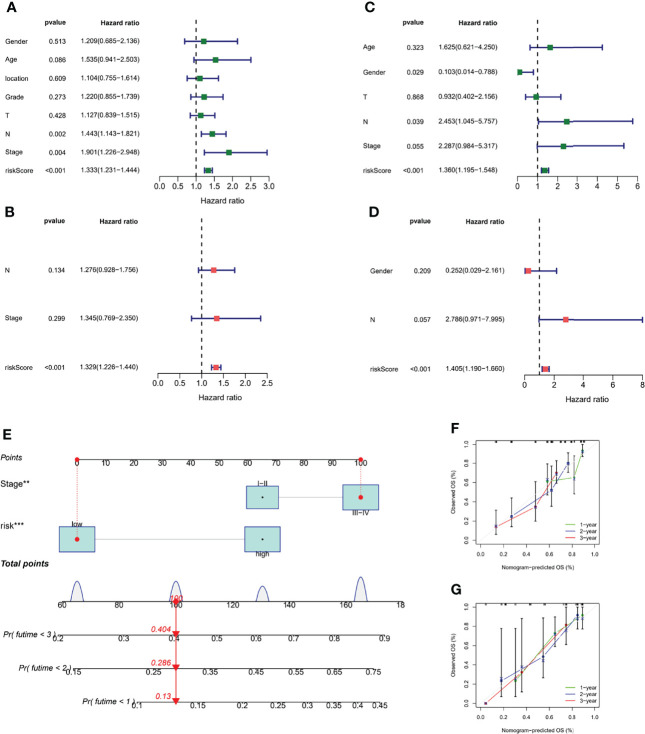
Validation of the m7G-LPS and construction of the nomogram. The univariate Cox forest map of risk score and clinical features in the GEO cohort **(A)** and TCGA cohort **(C)**. The multivariate Cox forest plot of risk score and clinical characteristics in the GEO cohort **(B)** and TCGA cohort **(D)**. **(E)** Construction of the nomogram based on risk score and stage. Calibration plots of the nomogram for the prediction of overall survival at 1, 2, and 3 years in the GEO cohort **(F)** and TCGA cohort **(G)**. **P < 0.01, ***P < 0.001.

To determine whether risk score was an independent prognostic factor for ESCC patients, we performed Cox regression analysis using clinical characteristics and risk score. Based on the results of univariate Cox regression analysis, there was a more significant correlation between risk score and OS in the TCGA and GEO cohorts [GEO cohort: HR = 1.333, 95% CI = 1.231-1.444, *p* < 0.001, **Figure 7**; TCGA cohort: HR = 1.360, 95% CI = 1.195-1.548, *p* < 0.001, **Figure 7**]. After adjusting for other confounding factors, risk score was an independent predictor of OS in ESCC patients [GEO cohort: HR = 1.329, 95% CI =1.226-1.440, *p* < 0.001, **Figure 7B** **;** TCGA cohort: HR = 1.405, 95% CI = 1.190-1.660, *p* < 0.001, **Figure 7**]. The nomogram can be visually applied to clinical work, as shown in **Figure 7E**. The calibration curves showed that the predicted curves were close to the standard curves in both cohorts, indicating that the predicted survival at 1, 2, and 3 years was highly consistent with the actual survival (**Figures 7F, G**). In addition, we also found a significant correlation between different risk subgroups and age, and tumor grade (*p* < 0.05, **Figure S2**).

### Comprehensive Analysis of Different Risk Subgroups

**Figure 8:**
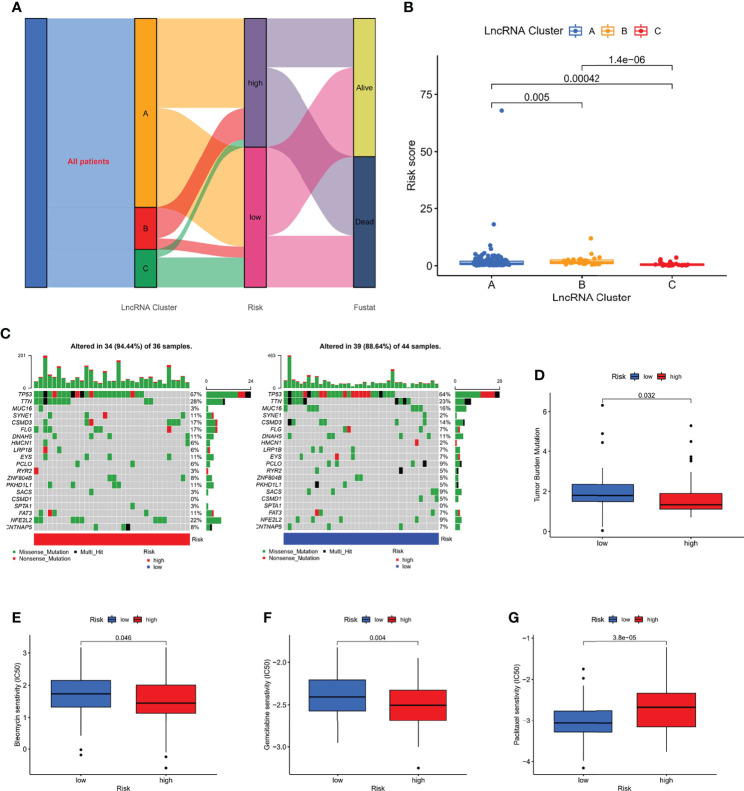
Correlation of risk score with molecular clusters, gene mutations, and chemotherapy sensitivity. **(A)** The sankey diagram of the relationship among the clusters, risk score and survival state. **(B)** Risk score levels of A/B/C clusters. **(C, D)** The waterfall plot of somatic mutation features in different risk subgroups. **(E–G)** The boxplot of Sensitivity of bleomycin, gemcitabine, paclitaxel in patients with high-risk subgroup and low-risk subgroup.

To explore the association between molecular clusters and risk groupings, we plotted sankey diagram and found that most patients with a poor prognosis in molecular clusters belonged to the high-risk group (**Figure 8A**). The box line plot confirmed our speculation that cluster A has the highest risk score, followed by cluster B (**Figure 8B**). We further analyzed the relationship between risk score and tumor mutation burden (TMB). Waterfall plots showed that high-risk patients exhibited a more extensive TMB than low-risk patients (**Figure 8C**). In addition, the box plot showed that low-risk patients had higher TMB scores (**Figure 8D**). In addition, we used the pRRophetic algorithm to evaluate the therapeutic effect of drugs. The IC50 results were encouraging:the high-risk group showed better efficacy for bleomycin (**Figure 8E**) and gemcitabine (**Figure 8F**) than the low-risk group, while the low-risk group showed better efficacy for paclitaxel (**Figure 8G**) than the high-risk group, suggesting that risk grouping based on risk score plays a guiding role in chemotherapy. In addition, the results of GSEA analysis suggested us that different risk subgroups may have different pathway activation states (**Figure S3**).

### Immunological Characteristics of Different Risk Subgroups

**Figure 9:**
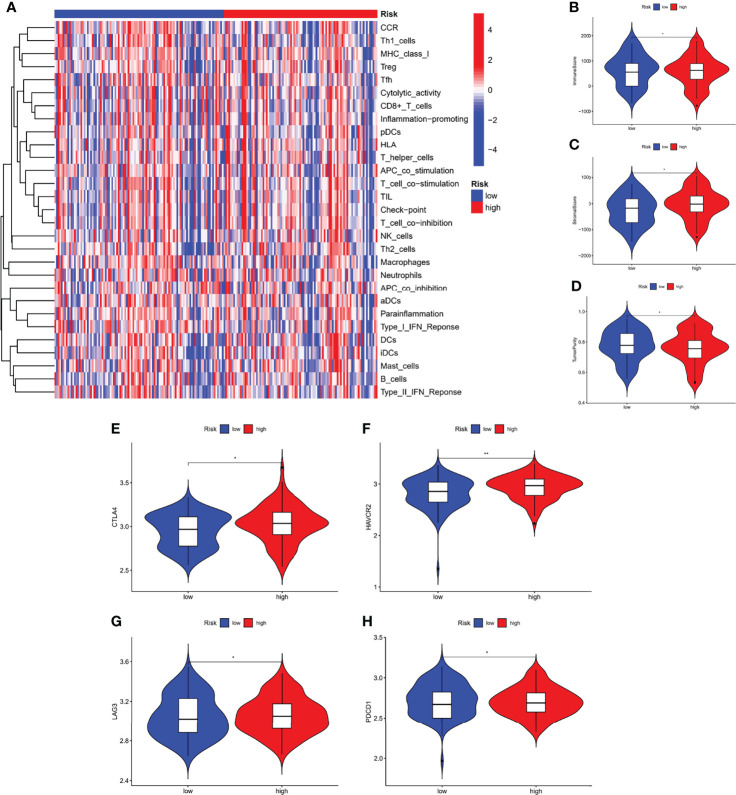
Immunological characteristics of different risk subgroups. **(A)** Heat map of differences in immune cell infiltration between different risk subgroups. The expression levels of the immune score **(B)**, stromal score **(C)**, and tumor purity **(D)**, between different risk subgroups. **(E–H)** The expression levels of immune checkpoint genes between different risk subgroups. *P < 0.05, **P < 0.01.

We performed an immune characterization analysis in the same way as described above for patients in different risk subgroups, and patients in the high-risk group had a higher degree of infiltration (**Figure 9A**). The high-risk group in the ESTIMATE analysis had higher stromal and immune scores and lower tumor purity (**Figures 9B–D**). Finally, we found that the four immune checkpoint mRNA expressions were equally different in the high- and low-risk subgroups (**Figures 9E–H**), possibly suggesting that high-risk group may benefit more from immunotherapy.

### Expression and Validation of m7G-related LncRNA

**Figure 10:**
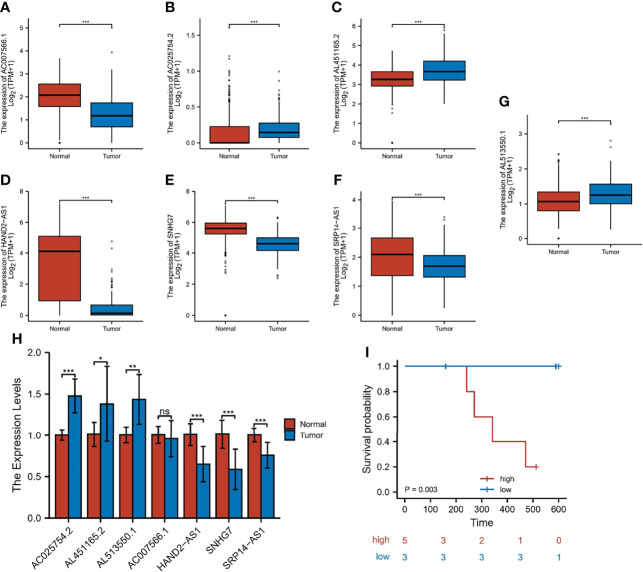
Expression and validation of 7 m7G-lncRNAs. **(A–G)** The differential expression of 7 m7G-lncRNAs in ESCC samples compared to adjacent normal controls in TCGA and GTEx RNA-seq data. **(H)** RT-qPCR of the expression of the 7 m7G-lncRNAs in 10 pairs of clinical samples. **(I)** Risk grouping and survival difference analysis in 8 patients. *P < 0.05, **P < 0.01, ***P < 0.001, ns, not significant.

To verify the expression of m7G-related lncRNA, we downloaded the normal human esophageal squamous epithelium sequencing set in the GTEx database and controlled it against TCGA-ESCC. The results indicated that the expression level of AC025754.2, AL451165.2, and AL513550.1 were significantly increased in ESCC tissue compared with normal adjacent tissue, whereas HAND2-AS1, SNHG7, SRP14-AS1, and AC007566.1 were downregulated in ESCC tissue (**Figure 10A**). We performed the qRT-PCR to detect the mRNA level of seven fundamental genes of our risk-score model in 8 patients’ fresh ESCC tissue and normal tissue. The expression difference of AC025754.2, AL451165.2, AL513550.1, HAND2-AS1, SNHG7, SRP14-AS1 between neoplastic and normal tissue in practical patients cohort were in accordance with that of TCGA and GTEx RNA-seq data (**Figure 10**). Collectively, these findings further validated the stability and reliability of the m7G-related lncRNAs prognostic signature. In addition, we grouped 8 patients with complete follow-up information in the 8 patients into high- and low-risk groups, with 5 being high-risk and 3 being low-risk, and their survival differences were statistically significant (**Figure 10I**).

## Discussion

Although there are many bioinformatics studies focus on the RNA modifications, to the best of our knowledge, this is the first analysis of the role of m7G-related lncRNAs in ESCC. We downloaded the data of 130 tumor samples from the GEO and TCGA databases to search for m7G-related lncRNAs. Twenty-one m7G-related genes were obtained from relevant literature as well as gene sets. We performed Spearman correlation analysis on 21 m7G-related genes and all annotable lncRNAs, and identified three molecular clusters by consensus clustering. Compared to patients with clusters A and C, patients with cluster B had a higher degree of immune cell infiltration and poorer OS. The characteristics of the TME also differed significantly between the three clusters. cluster B had the highest stromal and immune scores and the lowest tumor purity. cluster A had the lowest stromal and immune scores. and the highest tumor purity. The mRNA levels of immune checkpoints were higher in cluster B than in the other clusters. Furthermore, differences in survival states and immune landscapes between different clusters were significantly related to biological pathways. Thus, our findings indicate that m7G-related lncRNAs might serve as a predictor for evaluating the clinical outcome and immunotherapy response of ESCC.

To improve the accuracy and efficacy on predicting outcome, we constructed the robust and effective m7G-LPS based on 7 m7G-related lncRNAs and demonstrated its prognostic value and predictive ability in the GEO and TCGA cohorts. The m7G-LPS was able to serve as an independent risk factor and its risk score showed better prediction than the traditional clinical risk factors. The establishment of nomogram further improved the performance of m7G-LPS and facilitated its clinical application. Patients with better and worse prognosis in molecular typing showed lower and higher risk scores, respectively. Patients with low- and high-risk scores showed significantly different clinicopathological characteristics, prognosis, mutation, TME, immune checkpoints, and drug susceptibility.

Seven m7G-related lncRNAs were obtained, which were AC025754.2, AL451165.2, AL513550.1, AC007566.1, HAND2-AS1, SNHG7 and SRP14-AS1. LncRNA HAND2-AS1 exists on human chromosome 4 and mouse chromosome 8. Recent studies have shown that lncRNA HAND2-AS1 has an inhibitory effect on tumor occurrence and development, and is reduced in expression in several tumors (12). In Jin et al. reported that lncRNA HAND2-AS1 expression was reduced in patients with cervical cancer compared to normal subjects and that it inhibited the proliferation, migration and invasion of cancer cells by downregulating ROCK1 (13). In a study by Min et al, patients with Triple-negative breast cancer (TNBC) had significantly lower plasma levels of lncRNA HAND2-AS1 and were able to act as oncogenes in TNBC by downregulating RUNX 2 to inhibit cancer cell proliferation compared to healthy controls (14). Small nucleolar RNA host gene 7 (SNHG7) is a novel oncogenic lncRNA located on chromosome 9q34.3, which is 2,176 bp long (15). Recent studies have shown that SNHG7 promotes proliferation, migration and invasion of various cancers and inhibits tumor cell apoptosis (16, 17). For example, Zhang (18) et al. found that lncRNA SNHG7 was highly expressed in gastric cancer tissues and was closely associated with the proliferation, apoptosis, invasion and metastasis of gastric cancer cells. Among the 7 candidate lncRNAs, only HAND2-AS1 and SNHG7 had been reported as prognostic factors in ESCC (19, 20); the rest of lncRNAs were identified as prognostic signatures in BC for the first time.

The TME has a vital regulatory effect on carcinogenesis and tumor progression (21). Furthermore, lncRNAs and m7G are known to play a vital role in the TME (22, 23). Based on our scoring system, the immune scores and stromal score in the high-risk group were considerably greater than those in the low-risk group, although tumor purity exhibited the reverse tendency, which may explain the higher survival of patients in the low-risk group. Our findings aligned with those reported by Zeng et al. (24), indicating that patients with low immune scores show better OS than patients with high immune scores. ESCC is regarded as an immunogenic tumor. Nevertheless, to a great extent, immune dysfunction is mediated by inducing immunosuppressive cells to infiltrate the TME (25). For this purpose, we compared tumor-infiltrating immune cells among different subgroups, which serve as a powerful indicator to assess the tumor immune microenvironment. Similarly, immune infiltration cells were increased in the high-risk group compared with the low-risk group.

Numerous clinical trials evaluating the role of immune checkpoint inhibitors (ICIs) in patients with ESCC are currently in progress. By examining the association between the risk score and expression of critical immune checkpoints, it was further observed that most immune checkpoints (4/4) exhibited higher expression in the high-risk group. Therefore, patients with high-risk scores might benefit more from ICIs compared to patients with low-risk scores.

Chemotherapy remains the first-line treatment for advanced and metastatic ESCC. Owing to tumor heterogeneity in ESCC, the ESCC cases display variable sensitivity to chemotherapy (26). Therefore, we assessed the predictive value of the prognostic model for chemo-sensitivity in patients with ESCC. The IC50 results were encouraging:the high-risk group showed better efficacy for bleomycin and gemcitabine than the low-risk group, while the low-risk group showed better efficacy for paclitaxel than the high-risk group. Therefore, our prognostic model may function as a promising predictor of chemotherapeutic efficacy and may help in identifying the most appropriate chemotherapy regimen for each with ESCC.

Finally, we downloaded the normal human esophageal squamous epithelium sequencing set in the GTEx database and controlled it against TCGA-ESCC. In addition, we used RT-qPCR to detect the expression of the corresponding lncRNAs in 10 pairs of ESCC and adjacent tissues in our hospital. The results showed that among the lncRNAs involved in the modeling, our samples had the same expression trend as the public database samples except for AC007566.1. Furthermore, we grouped 8 patients with complete follow-up information in the 10 samples into high- and low-risk groups, with 5 being high-risk and 3 being low-risk, and their survival differences were statistically significant.

## Conclusion

In summary, this study systematically assessed the prognostic value, role in the TME, and potential regulatory mechanisms of m7G-lncRNAs in ESCC. We identified three ESCC clusters via consensus clustering and developed a m7G-LPS based on m7G-related lncRNA profiles of ESCC, which stratified the prognosis and presented the significantly different TME. This is the first study to reveal that m7G-lncRNAs play a vital role in the prognosis and TME of ESCC.

## Data Availability Statement

The original contributions presented in the study are included in the article/**Supplementary Material**. Further inquiries can be directed to the corresponding author.

## Ethics Statement

The studies involving human participants were reviewed and approved by The Ethics Committee of the Second Hospital of Hebei Medical University. The patients/participants provided their written informed consent to participate in this study.

## Author Contributions

FZ and SL conceived and designed the study. ZD, YL, and SL collected and analyzed the data. PG and DZ wrote the manuscript. All authors read and approved the manuscript.

## Funding

This study was supported by Hebei Provincial Government sponsored the project of training excellent talents in clinical medicine (No. 303-2022-27-37).

## Conflict of Interest

The authors declare that the research was conducted in the absence of any commercial or financial relationships that could be construed as a potential conflict of interest.

## Publisher’s Note

All claims expressed in this article are solely those of the authors and do not necessarily represent those of their affiliated organizations, or those of the publisher, the editors and the reviewers. Any product that may be evaluated in this article, or claim that may be made by its manufacturer, is not guaranteed or endorsed by the publisher.

## Supplementary Material

Supplementary Figure 1 The overall workflow of the current study.

Supplementary Figure 2 Correlation of the high-/low-risk subgroups with different clinical characteristics. The heatmap showed associations between the expression of the 7 m7G-related lncRNAs in the low- and high-risk group and clinicopathological features in the GEO cohort **(A)** and TCGA cohort **(B)**. **P* < 0.05, ***P* < 0.01.

Supplementary Figure 3 The KEGG signaling pathways in different risk subgroups.
