# Supplementary figures and images for: Comprehensive Analysis of Molecular Clusters and Prognostic Signature Based on m7G-related LncRNAs in Esophageal Squamous Cell Carcinoma
Source: Front Oncol. 2022 Jul 14;12:893186. doi: 10.3389/fonc.2022.893186 (PMC9329704; doi:10.3389/fonc.2022.893186)

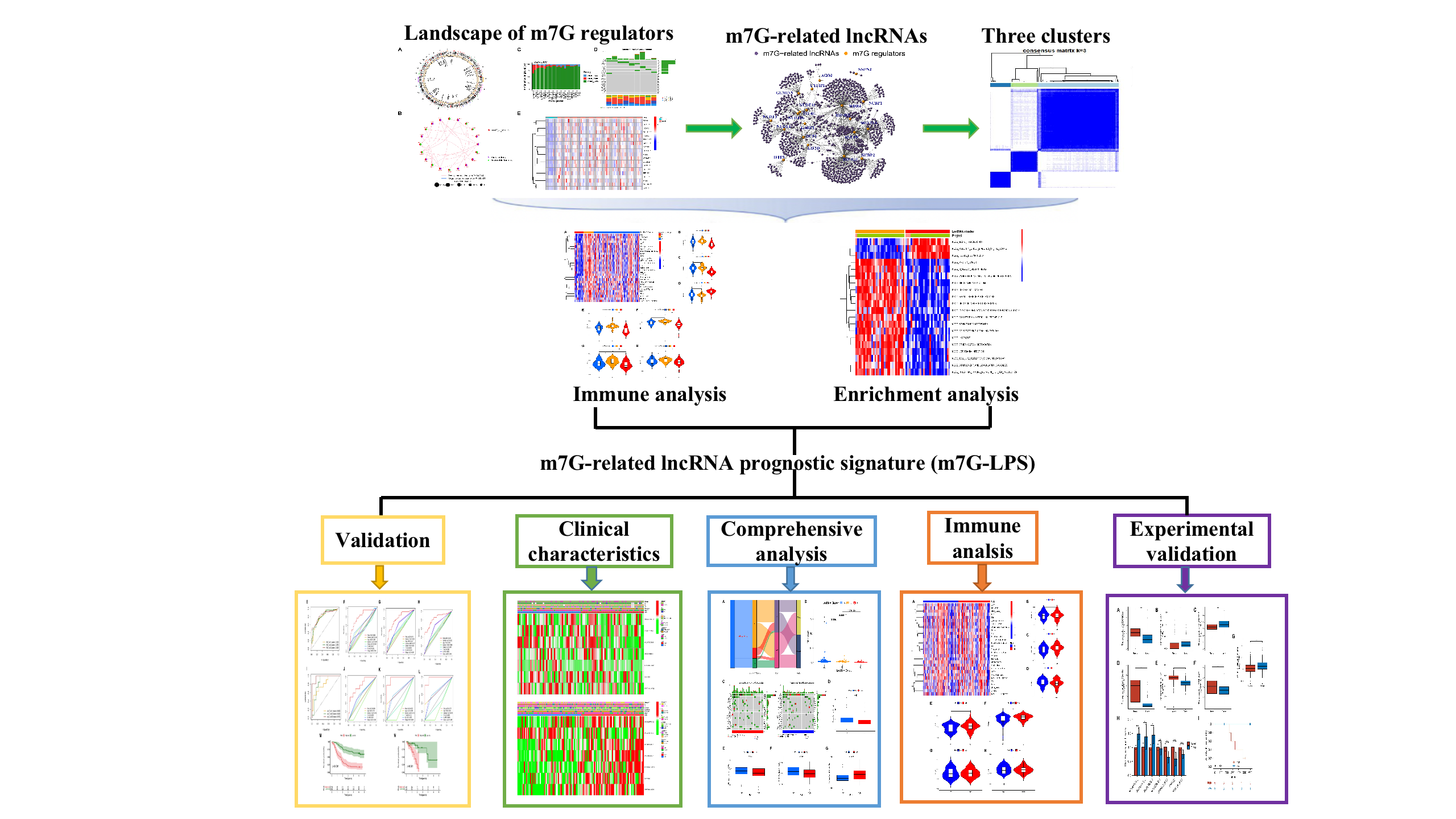

Supplement: Supplementary Figure 1 — The overall workflow of the current study. [file Image_1.tif]

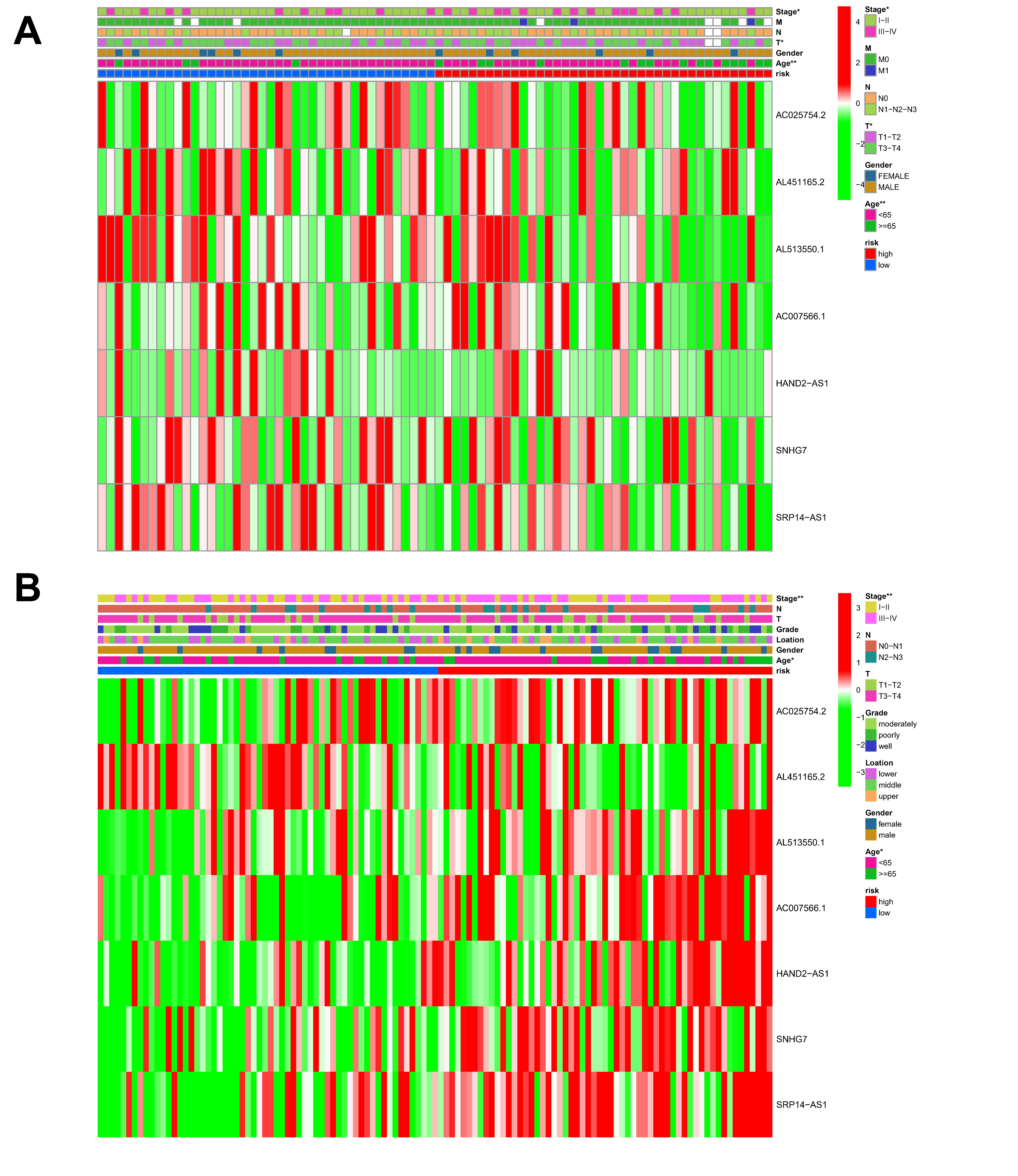

Supplement: Supplementary Figure 2 — Correlation of the high-/low-risk subgroups with different clinical characteristics. The heatmap showed associations between the expression of the 7 m7G-related lncRNAs in the low- and high-risk group and clinicopathological features in the GEO cohort (A) and TCGA cohort (B). *P < 0.05, **P < 0.01. [file Image_2.tiff]

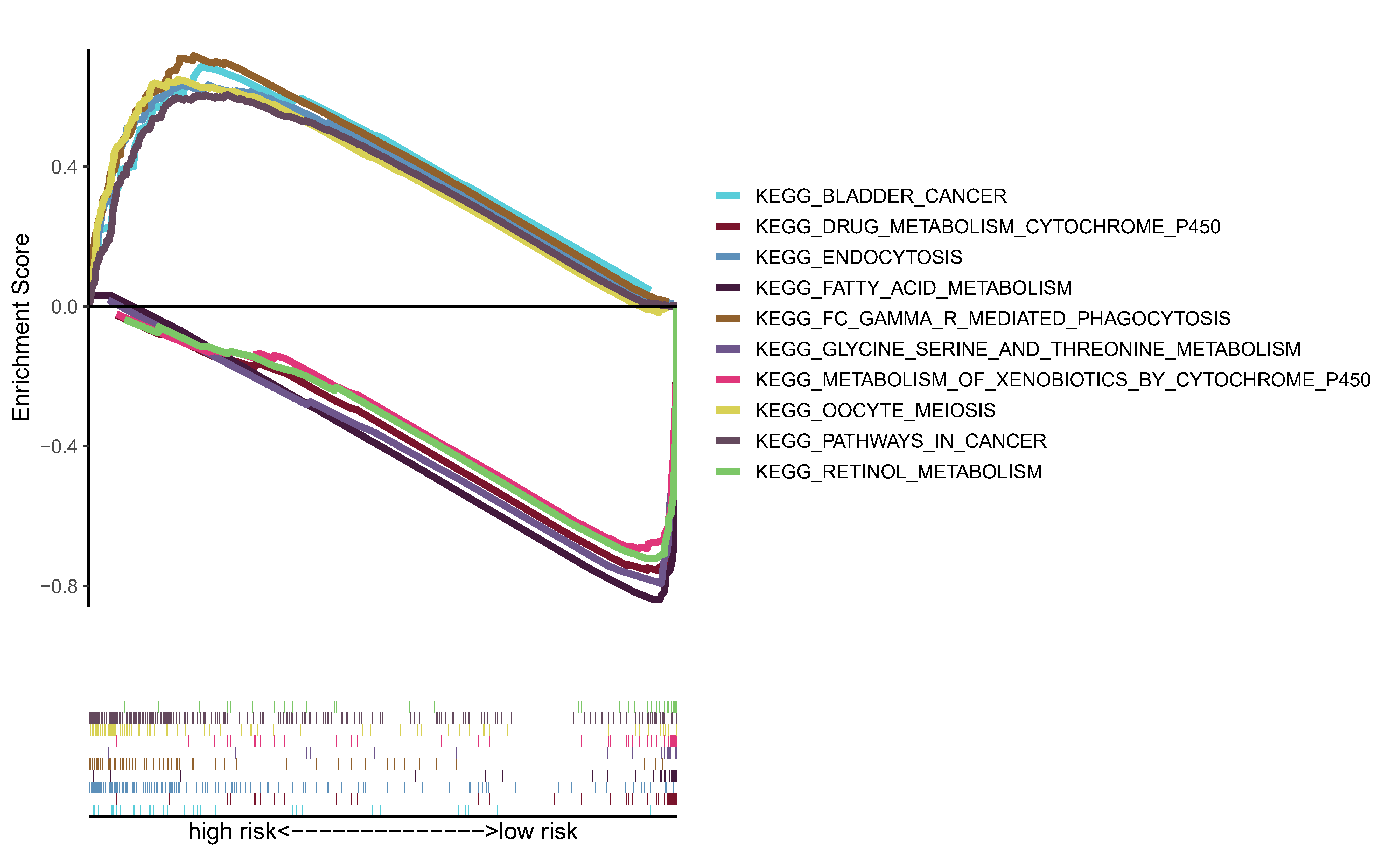

Supplement: Supplementary Figure 3 — The KEGG signaling pathways in different risk subgroups. [file Image_3.tiff]
